# The Neurocircuitry Underlying Additive Effects of Safety Instruction on Extinction Learning

**DOI:** 10.3389/fnbeh.2020.576247

**Published:** 2021-01-12

**Authors:** Arash Javanbakht, Lana Ruvolo Grasser, Shantanu Madaboosi, Asadur Chowdury, Israel Liberzon, Vaibhav A. Diwadkar

**Affiliations:** ^1^Department of Psychiatry and Behavioral Neurosciences, Wayne State University School of Medicine, Detroit, MI, United States; ^2^Department of Psychiatry, Texas A&M University Central Texas, Killeen, TX, United States

**Keywords:** instructed extinction, extinction learning, fear conditioning, fear extinction, informed extinction, fMRI, PPI

## Abstract

Extinction learning is the dominant laboratory model for exposure therapy, a treatment involving both experience of safety near the feared object, and safety instructions relayed by a therapist. While the experiential aspect of extinction learning is well researched, less is known about instructed extinction learning and its neurocircuitry. Here, in 14 healthy participants we examined the neural correlates of, and the network interactions evoked by instructed extinction learning. Following fear conditioning to two CS+ stimuli, participants were instructed about the absence of the aversive unconditioned stimulus (US) for one of the CS+s (instructed CS; CS+I) but not the second CS+ (uninstructed CS+; CS+U). Early during extinction learning, greater activation was observed for the CS+I > CS+U contrast in regions including the vmPFC, dmPFC, vlPFC, and right parahippocampus. Subsequently, psychophysiological interaction (PPI) was applied to investigate functional connectivity of a seed in the vmPFC. This analyses revealed significant modulation of the dmPFC, parahippocampus, amygdala, and insula. Our findings suggest that the addition of cognitive instruction yields greater activation of emotion regulation and reappraisal networks during extinction learning. This work is a step in advancing laboratory paradigms that more accurately model exposure therapy and identifies regions which may be potential targets for neuromodulation to enhance psychotherapy effects.

## Introduction

Fear conditioning is the established laboratory model for emotional learning (Armony et al., 1997; Craske et al., 2014). During classical fear conditioning, a neutral cue (conditioned stimulus, CS+) is repeatedly paired with an aversive stimulus (unconditioned stimulus, US), leading to the development of a fear response to the CS+ (even in the absence of the US) termed the conditioned response (CR). The CR may be defined as increased startle response, elevated electrodermal activity, and/or increased self-reported fear/anxiety/expectation of the CS-US pairing (Grasser and Jovanovic, 2020). This self-reported knowledge that an individual may have regarding the CS predicting the US is called contingency awareness (Craske et al., 2008), and contingency awareness in turn is a strong correlate of the conditioned response (Purkis and Lipp, 2007). Extinction learning is a complement to fear conditioning in which a new competing memory that is formed indicates that the CS+ is no longer predictive of the US. This is achieved via repeated presentation of the CS+ without the US, leading to a decay in the CR (Milad and Quirk, 2012). Extinction learning is a form of safety learning (Grasser and Jovanovic, 2020) and a key mechanism (and thus a dominant laboratory model) for exposure therapy (Craske et al., 2008). Notably, abnormalities in extinction learning have been linked to fear related disorders such as phobias and post-traumatic stress disorder (Norrholm and Jovanovic, 2018).

Fear and safety learning in humans occur through a combination of direct experience (Pavlovian conditioning), observation (of others being exposed to adverse and safe events), and instruction (Olsson and Phelps, 2004). Instruction-mediated conditioning and learning is highly compelling from the perspective of top-down cortical mechanisms and relevant from the perspective of exposure therapy (Javanbakht et al., 2017). Behavioral and psychophysiology studies have demonstrated that in addition to fear conditioning solely through direct experience (as would most likely happen naturalistically), explicitly instructing participants to expect the US to follow the CS+ also leads to the development of a fear response to the CS+ (Olsson and Phelps, 2004; Raes et al., 2014; Cameron et al., 2016). The aforementioned studies have found that Pavlovian, instructed, and observational methods of fear conditioning result in similar levels of learning. In a similar vein, participants can be instructed about the *absence* of previously established contingency relationships between the CS+ and the US, thereby motivating extinction learning solely through instruction. For example, when instructed that they will no longer receive an aversive US after a previously conditioned CS+, participants show either immediate extinction (Mower, 1938; Rowles et al., 2012; Sevenster et al., 2012), or faster decay of the fear response (Koenig and Henriksen, 2005). These studies have provided valuable information regarding different learning mechanisms through which fear can be extinguished, but they have been limited by (a) between-subjects designs and (b) lack of neuroimaging data.

Complementary “bottom-up” and “top-down” mechanisms mediate brain network interactions underpinning higher-level processes such as self-referential processing (Frewen et al., 2020). “Bottom-up” mechanisms may primarily engage the salience network [insula, anterior cingulate cortex (ACC), and amygdala—see below] to alert individuals to threatening and rewarding stimuli in the environment without conscious knowledge/action. “Top-down” mechanisms may primarily engage the prefrontal cortex to consciously select and attend to stimuli in the environment based on conscious will/effort, and to regulate reactive “bottom-up” responses. Both are presumed to in part underpin psychotherapeutic efficacy (Malejko et al., 2017). Thus, studying their combined effects can inform neuroscientific theories about fear and safety, and enhance the clinical relevance of laboratory models (Javanbakht et al., 2017). In this work, we experimentally manipulated experience and instruction-based extinction learning, using an event-related fMRI design with the aim of identifying the evoked brain network profiles.

A comprehensive overview of the known neurocircuitry of fear learning is beyond our scope (Maren, 2001). Nevertheless, we succinctly sample from a wealth of neuroimaging data to provide a brief summary of key regions herein. The regions in this circuit include the dorsal anterior insula, dorsal ACC, and the amygdala (Adolphs, 2013; Yin et al., 2018), all of which comprise the salience network. The amygdala also playing a significant role in the development of extinction learning, disseminating signals across cortex, including throughout prefrontal regions, to orient and alert the brain to salient stimuli in the environment. Moreover, the structure activates both brainstem and hypothalamus to mount behavioral responses via the sympathetic adrenal medullary axis and the hypothalamic pituitary adrenal axis (Ross et al., 2017). The hippocampus and prefrontal cortex are involved in the formation and recall of extinction memories via context processing, consolidation, and retrieval (Greco and Liberzon, 2016), and the ventromedial prefrontal cortex (vmPFC) has a specific regulatory function regarding the inhibition of the amygdala and the fear response during extinction learning (Kim et al., 2003; Urry et al., 2006; Milad et al., 2007b; Ganella et al., 2017). Both the hippocampus and vmPFC are anatomically connected to the amygdala (Corches et al., 2019). Other areas involved in extinction learning include the anterior cingulate and dorsolateral prefrontal cortex (Greco and Liberzon, 2016). Preclinical studies (Fendt, 1998, 2000; Meyer et al., 2019) corroborate the findings from *in vivo* neuroimaging work in humans.

Functional imaging studies of *instructed* extinction learning are absent, though some studies have attempted to understand the neuronal bases of observational learning. For example, the ventromedial prefrontal cortex (vmPFC) is involved in vicarious learning of safety via observing others (Golkar et al., 2016) and, along with the hippocampus, context processing during extinction learning (Ahs et al., 2015; Hermann et al., 2017). *Context* plays a key role in extinction learning (Bouton, 2002; Bouton et al., 2006). Most studies focus primarily on *physical* context, but in fact context covers a broader spectrum including internal, temporal, and social/cognitive components (Bouton, 2002; Bouton et al., 2006; Liberzon and Sripada, 2008; Javanbakht, 2018). In this spectrum, *cognitive* context (operationalized as explicit instruction) may recruit the hippocampus and prefrontal cortex to judge the relevance of stimuli to memories and strategic goals (Liberzon and Sripada, 2008). Interestingly, instructed reappraisal, another method of emotion regulation, also activates the vmPFC as well as the dmPFC, the dlPFC, and the ACC (Pico-Perez et al., 2019).

In a previous behavioral study (Javanbakht et al., 2017) we examined the additive effects of safety instruction and experience on extinction learning. In the conditioning phase, participants were conditioned to two CS+ stimuli. Then, prior to extinction, they were instructed that the contingency pairing would be removed from one CS+, US contingency. The study revealed that instruction elicited a smaller fear response (measured by skin conductance) compared to the uninstructed CS+ during the *early* phase of extinction learning. These findings were the first to demonstrate the salient effects of instruction in facilitating extinction learning using a within-subject design comparing instruction + experience, vs. experience alone, but it did not investigate the induced functional interactions within the neurocircuitry of fear.

In the present investigation and analyses, we explore how the combined effects of safety instruction and experiential learning modulated the (a) activation of brain regions and (b) network profiles in the brain’s fear circuit. Notably, we hypothesized that the additive effect of instruction with experiential extinction learning would recruit the vmPFC and hippocampus, brain areas involved in emotion regulation and context processing. Psychophysiological interaction (PPI) was used as a simple framework for estimating directional (i.e., from seed-to-target) functional connectivity between a seed region (which in our case was the vmPFC) and its potential functional targets (O’Reilly et al., 2012; Silverstein et al., 2016).

## Materials and Methods

### Participants

Thirty-seven (37) healthy male (*n* = 17) and female (*n* = 20) participants between the ages of 18 and 45 (${\bar{X}}_{\text{age}}$ = 26.18, *SD* = 4.61) were recruited for this study using approved flyers and university forum posts. Oral consent and initial eligibility screening were completed via phone interview. Six participants were excluded due to excess motion (> 4 mm) during MR imaging, leaving 31 (male = 16, female = 15, ${\bar{X}}_{\text{age}}$ = 26.18, *SD* = 4.78) participants with usable data to include for analysis. The Institutional Review Board at WSU approved the protocol and all procedures herein. All participants gave informed consent to participate in the study and were able to tolerate small, enclosed spaces associated with fMRI data acquisition without anxiety. Exclusion criteria were: (1) lifetime psychiatric diagnoses (with the exception of history of substance related disorders more than 1 year prior), (2) serious medical or neurological illness that could compromise brain function, (3) history of significant closed head injury, (4) metal, implants, or metallic substances in the body, and/or (5) pregnant or trying to become pregnant. The Mini-International Neuropsychiatric Interview (MINI) was used to rule out psychiatric diagnoses. Consent and administration of the MINI were concurrent, on a separate screening day from the neuroimaging scans (and up to 2 weeks in advance of MRI acquisition). At the time of the screening, participants were exposed to a single trial of the US, a white noise burst described below, to ensure that they could tolerate the sound.

### fMRI

Multiband gradient echo EPI fMRI was conducted on a 3T Siemens Verio system using a 32-channel volume head coil (310 vol, TR = 2 s, TE = 29 ms, multiband factor = 3, FOV = 256 × 256 × 144 mm^3^, acquisition matrix = 128 × 128, 72 axial slices, pixel resolution = 2 × 2 × 2 mm^3^, 10:48 mts). A high-resolution (1 mm^3^) structural T_1_-weighted MRI image was also collected. A scout image in each plane was acquired followed by a 3D T_1_-weighted anatomical MRI image [3D Magnetization Prepared Rapid Gradient Echo (MPRAGE) sequence, TR = 2,150 ms, TE = 3.53 ms, TI-1,100 ms, flip-angle = 8°, FOV = 256 × 256 × 160 mm^3^, 160 axial slices of thickness = 1 mm, pixel resolution = 1 × 1 × 1 mm^3^, 4:59 mts].

### Fear Conditioning and Extinction Learning Paradigm

**FIGURE 1:**
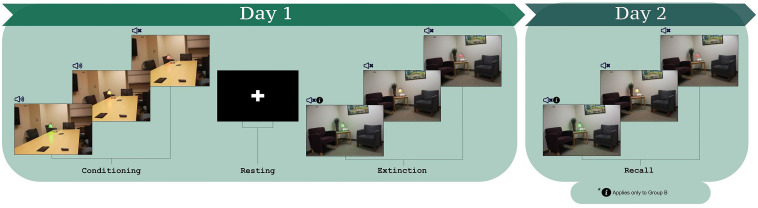
The fear conditioning and extinction learning paradigm (Milad et al., 2005, 2007a,b, 2008, 2009; Milad and Quirk, 2012; Marin et al., 2017; Raij et al., 2018). Participants learned the CS-US contingencies during conditioning. During extinction learning, participants were instructed that for one of the CS+s, they would not hear the loud noise (CS+I). For the other CS+, no information was provided (CS+U). Three lamp colors comprise the three CSs, which were counterbalanced within and across participants as CS+I, CS+U, and CS–.

The employed paradigm was identical to one used previously in a study of instructed fear and extinction learning (Javanbakht et al., 2017) and has been generally applied to study fear and extinction learning (Milad et al., 2005, 2007a,b, 2008, 2009; Milad and Quirk, 2012; Marin et al., 2017; Raij et al., 2018). During the task, stimuli were presented via an MR compatible projector with the paradigm controlled using ePrime 2.0 (Schneider et al., 2002). CSs included pictures of a lamp with three different colors (red, green, and yellow) placed either in an office or in a conference room (stimuli presented in Figure 1). The different rooms provided context such that one room was presented consistently during conditioning and the other room during extinction (Milad et al., 2005; Marin et al., 2017; Raij et al., 2018). The assignment of lamp colors and context images to participants was randomized and counterbalanced (randomly differed across participants). The US was a 95 db white noise burst (presented for 500 ms) (Sperl et al., 2016) conveyed through MRI compatible noise canceling headphones. The headphones were worn during conditioning and extinction, signaling the possibility of the US being administered throughout all phases.

The protocol consisted of habituation, conditioning, and extinction learning (protocol schematic in Figure 1; Milad et al., 2005, 2007a,b, 2008, 2009; Milad and Quirk, 2012; Javanbakht et al., 2017; Marin et al., 2017; Raij et al., 2018). For habituation, participants were informed that they would see a series of images, none of which would be followed by a loud noise. The purpose of the habituation phase is to remove the effects of novelty of the images that will be presented during the task. Participants saw each of the three CSs, once in the fear conditioning context and once in the extinction context, over four trials, and never heard the loud noise. Fear conditioning immediately followed. Participants were told that they would see the previous images, some of which would be followed by a loud noise. Each image was presented for 4 s with jittered inter-trial intervals between 6 and 12 s. Each CS was presented 15 times; the two CS+ stimuli were paired with the US for 10 trials (i.e., 66% reinforcement). The US was presented 3.5 s after CS+ onset. The third CS (CS-) was never paired with the US.

The session concluded with extinction learning, 10 min after fear conditioning. Prior to extinction learning, all participants were presented with instructions stating that they would *not* hear the loud noise when they saw one of the two CS+s (Instructed CS, CS+I, “You will not hear the loud noise with the red light”). The other CS+ was defined as the uninstructed CS+, CS+U. For extinction learning, each CS was presented 12 times for 4 s with jittered inter-trial intervals between 6 and 12 s, without the US.

At the end of the conditioning phase and again at the end of the extinction phase, participants were asked to verbally rate how much they expected to hear the loud noise when presented with each CS on a scale of 1–5, with 1 being “Not at All” and 5 being “Very Much So” (5-point likert scale). This verbally reported expectancy data was acquired for both CS+s and the CS−. Contingency awareness was defined based on expectancy data from immediately following the end of conditioning as the average of the two ratings for CS+I and CS+U being greater than that of the rating for the CS− (Tabbert et al., 2006; Javanbakht et al., 2017). Average rating for the CS+s equal to or less than the rating for the CS− was the threshold for exclusion/classification of being “unaware.” Those who were not classified as “aware” of the CS-US contingency (“unaware” participants) were subsequently removed from the present analysis (Tabbert et al., 2006; Javanbakht et al., 2017). Participants for which there was no evidence of learning the CS-US contingency (“unaware”) merited exclusion, given that conditioned fear must be established in order for extinction learning to be possible, and the goal of this study was to examine extinction learning.

### Data Analysis

#### Self-Reported Expectancy

As described above, participants were asked to verbally rate how much they expected to hear the loud noise when presented with each CS on a 5-point likert scale at the conclusion of each phase—conditioning and extinction. After conditioning phase, this self-report measure of contingency/expectancy awareness was used to determine whether participants learned the CS-US contingency. We also obtained psychophysiological recordings (skin conductance response) from participants while in the scanner, but were unable to collect a sufficient amount of viable data. Measuring skin conductance in a 3T static magnetic field is a challenge both due to interference with the MRI RF. The lack of viable skin conductance data is a common problem and inherent limitation, as such data are susceptible to motion and high levels of noise (Bjorkstrand, 1990). Self-report contingency awareness data allowed us to deem participants to be “aware” of the CS-US contingency (see above).

All data were checked for out-of-range values, normality, linearity, homoscedasticity, and outliers. To compare self-reported expectancy of CS’ by phase (conditioning and extinction) and CS type (CS+I, CS+U, and CS−), a 2 × 3 within-subjects repeated measures ANOVA was performed in SPSS. Mauchly’s test indicated the assumption of sphericity had been met. Main effects of phase, CS type, and phase × CS type were all compared using Bonferroni corrected pairwise comparisons with 95% confidence intervals.

#### fMRI Preprocessing and Statistical Modeling

fMRI data were preprocessed and analyzed using standard methods in MATLAB R2013b with the Statistical Parametric Mapping toolbox (SPM12). For spatial pre-processing, the structural images were manually oriented to the AC-PC line with the reorientation vector applied across EPI image sets. Structural images were then realigned to a reference image to correct for head movement and subsequently co-registered to the structural image. The high resolution T_1_ image was segmented and normalized to the MNI template, with the resultant deformations applied to the EPI image set. Low frequency components (scanner drift, physiological noises, etc.) were removed using a high-pass filter (128 s), and the EPI images were spatially smoothed using a Gaussian filter (8 mm full-width half maximum). Because of the previously observed difference between response to CS+I and CS+U during early extinction, and that at the end of the extinction CRs to both CSs fully extinguished (Javanbakht et al., 2017), we separated early extinction (first six trials of each CS+) from late extinction, as at the beginning of the extinction phase, individuals are learning that the CS+ is no longer associated with the US and forming a new, competing safety memory. At the end of the extinction phase, the assumption is (or at least the goal is to ensure) that a competing safety memory has been formed and the CR is extinguished. In first level analyses, events were modeled as regressors (duration 4 s box cars convolved with a canonical HRF) representing the CS+ (Early and Late), CS− (Early and Late) and inter-trial intervals (ITI). Per convention, an autoregressive AR(1) model was used to account for serial correlation, and the six motion parameters (three for translation and three for rotation) were included as effects of no interest. To examine the additive effects of instruction on activation profiles of extinction learning, regressors representing early phases of CS+I > CS+U were forwarded to a second level random effects model.

A region of interest (ROI) approach was employed, restricting analyses to regions involved in extinction learning and emotion processing (Greco and Liberzon, 2016) (see section “Introduction”). The ROI approach was implemented using deterministic masks in stereotactic space (Maldjian et al., 2003). The *a priori* set of regions included the bilateral ACC and ventral PFC, bilateral amygdala, bilateral dorsal PFC, bilateral hippocampus, bilateral insula, and parahippocampal gyrus. This hypothesis driven *a priori* ROI-based approach was motivated by prior knowledge of fear circuitry (see section “Introduction”).

Across all analyses, significant clusters were identified estimating the minimum cluster extent for activated voxels to be rejected as false positive (noise-only) clusters (Woodcock et al., 2016; Friedman et al., 2017). This approach performs a Monte Carlo alpha probability simulation, computing the probability of a random field of noise (after accounting for the spatial correlations of voxels based on the image smoothness within each region of interest estimated directly from the data set) to produce a cluster of a given size, after the noise is thresholded at a given level. Thus, instead of using the individual voxel probability (height) threshold alone in achieving the desired overall significance level, the method uses a combination of both probability and minimum cluster size thresholding.

## Results

Fifteen participants did not show evidence of the CS-US contingency awareness after conditioning (i.e., those who equally self-reported expected the US when presented with the CS+s and CS−, and those who indicated greater self-reported expectancy of the US when presented with the CS- than when presented with the CS+s). Because these participants were deemed to have failed the objective behavioral criteria for the experiment, they were excluded from the neuroimaging analyses. An additional six participants had to excluded on account of excess motion during the acquisition. Thus the final analyses included only those participants who did develop contingency awareness after conditioning *and* with viable neuroimaging data (*n* = 14).

Our subsequently presented results are organized as follows: (1) First, we present self-reported contingency awareness data for 14 participants, that reflect participant learning and task effects; (2) Next, we describe activation-based fMRI findings for each of the task conditions and comparisons (see section “Materials and Methods”); (3) These activation-based results motivated further analyses related to network effects, conducted using basic models of directional functional connectivity based on PPI (Friston et al., 1997; O’Reilly et al., 2012; Silverstein et al., 2016).

### Self-Reported Contingency Awareness

As noted, self-reported expectancy information was used as a measure of contingency awareness and successful conditioning (given the absence of physiological data). Psychophysiological measures (e.g., skin conductance) are limited by large inter-individual variability and the reality that some participants do not show measurable levels of SCR during the task (Rabinak et al., 2017). Self-reported expectancy ratings of CS-US contingencies have sufficient face, diagnostic, predictive, and constructive validity, warranting them apt to assess contingency awareness (Boddez et al., 2013; Rabinak et al., 2017).

**FIGURE 2:**
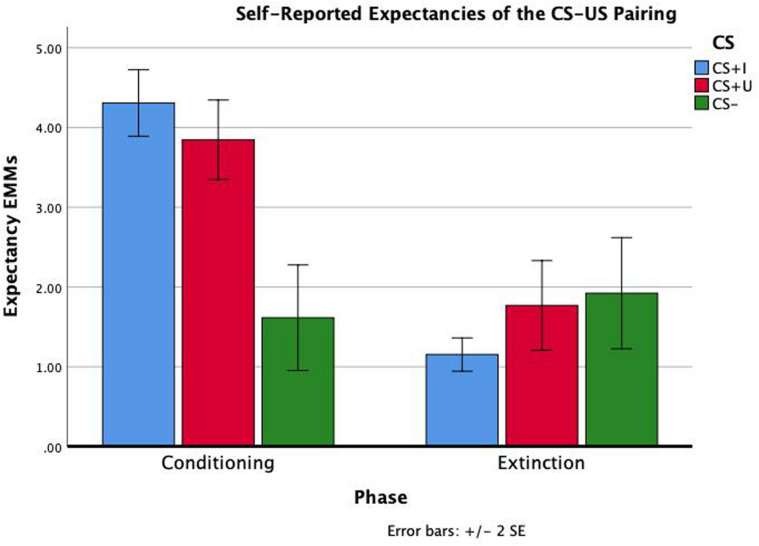
Repeated measure ANOVA of phase × CS type on self-reported expectancies of the CS-US contingency. Self-reported contingency information was obtained at the end of conditioning and the end of extinction for each CS. There were significant effects of phase, CS type, and phase × CS type (all *p*’s < 0.01). Estimated marginal means (EMMs) reflect the mean response for each factor, adjusted for the other variables in the model. Error bars ± 2*SE* of the mean. Test results, including *post hoc* comparisons, are all reported in Table 1.

Test statistics for the repeated measure ANOVA are reported in Table 1. The within subjects effects of phase, CS type, and phase × CS type were all significant (*p*s < 0.001). Bonferroni-corrected pairwise comparisons indicated that expectancies across CS type were significantly greater following conditioning compared to extinction (*p* < 0.001). Bonferroni-corrected pairwise comparisons also indicated that expectancies across phase were significantly greater for CS+I compared to CS− and CS+U compared to CS- (*p*’s = 0.001 and 0.003), but not CS+I compared to CS+U, *p* = 1.00. Therefore, the expectancy data indicated that participants had a greater expectancy of the CS-US contingency in after conditioning compared to after extinction learning and for both CS+I and CS+U compared to CS−. Participants’ self-reported expectancy data indicates that they conditioned to the CS+s equally but not the CS-during conditioning, and extinguished conditioned fear to both CS+s by the end of the extinction phase (see Figure 2).

**TABLE 1:** Test statistics for repeated measures ANOVA of self-reported expectancy of CS-US contingencies after conditioning and after extinction.

*Main effects*	*F*	*df*	Bonferroni corrected 95% CIs (LLCI, ULCI)	*P*-values
Phase	58.284	1,13	1.161, 2.077	<0.001*
CS type	14.980	2,26		<0.001*
CS+I v. CS+U			−0.567, 0.424	1.00
CS+I v. CS−			0.398, 1.530	0.001*
CS+U v. CS−			0.369, 1.703	0.003*
Phase × CS Type	29.922	2, 26		<0.001
Conditioning, CS+I			3.751, 4.677	*
Conditioning, CS+U			3.270, 4.301	*
Conditioning, CS−			0.903, 2.240	*
Extinction, CS+I			0.933, 1.353	*
Extinction, CS+U			1.140, 2.288	*
Extinction, CS−			1.146, 2.568	*

For significance interpretation of Phase × CS Type interaction effects, the test of a significant interaction effect was significant, p < 0.001, and Bonferroni corrected 95% confidence intervals (CIs) are reported for specific effects where CIs that do not cross 0 are considered to represent significant effects, which are marked with an *.

### Regional Activation Differences

**FIGURE 3:**
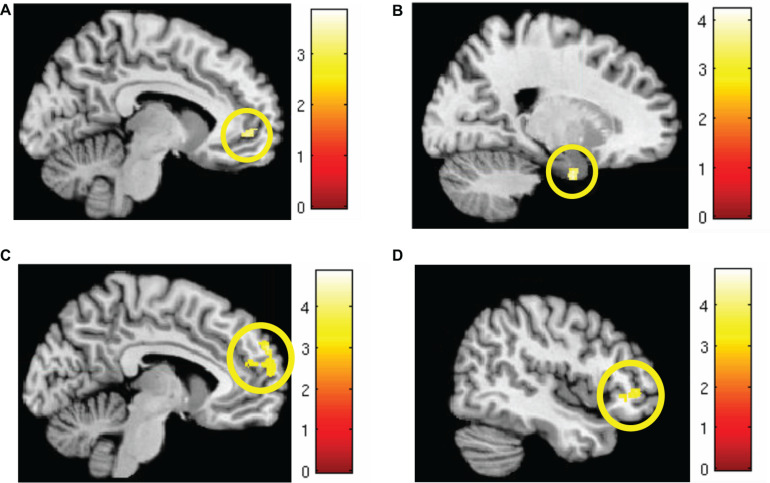
Areas of significant regional activation during the first 6 trials of the extinction learning phase, CS+I > CS+U. **(A)** Left vmPFC; **(B)** right parahippocampus; **(C)** left dmPFC; and **(D)** bilateral vlPFC. MC corrected *p* ≤ 0.001. Heat maps for each region represent *Z*-scores, which are reported alongside *p*-values in Table 3.

Markov chain Monte Carlo (MCMC) minimum cluster thresholds can be found in Table 2 for all bilateral masks, including those for which non-significant findings were not identified. During early *extinction* (first six trials), significant activation (CS+I > CS+U) was induced in the left vmPFC, left dmPFC, bilateral vlPFC, and right parahippocampus (Figure 3; test statistics available in Table 3), though no significant differences were observed in the converse contrast of CS+U > CS+I. Based on previous studies, our hypothesis, and our finding of involvement of vmPFC, this node was employed as an *a priori* seed in subsequent exploratory PPI analyses.

**TABLE 2:** Minimum cluster thresholds by mask for Monte Carlo corrections for multiple comparisons (MCMC) simulations.

*Early extinction, CS+I > CS+U*	MCMC minimum cluster threshold (regional activation; *p* ≤ 0.005)	MCMC minimum cluster threshold (vmPFC interaction; *p* ≤ 0.05)
Anterior cingulate cortex and ventral prefrontal cortex	55.6	20.5
Amgydala	15.2	34.9
Dorsal prefrontal cortex	173	87.3
Hippocampus	33.4	42.5
Insula	38.7	44.2
Parahippocampus	28.9	35.4

**Minimum cluster thresholds represent the minimum required number of voxels showing significant activation greater than that due to noise at the a priori established p-value of 0.005 for the ROI-based analysis and 0.05 for PPI with the left vmPFC as the seed. All masks, including those for which there were no significant findings, are presented. Masks are bilateral; contrast is CS+I > CS+U for the early phase (first six trials) of extinction.**

**TABLE 3:** Significant regions of interest activated during extinction learning.

*Early extinction, CS+I > CS+U*	Cluster size corrected in voxels	Coordinates	*P*-values (peak)	*Z*-scores
Left vmPFC	81	−10, 50, −1	0.001	3.09
Right parahippocampus	54	20, −1, −30	0.001	3.29
Left dmPFC	432	−9, 60, 29	<0.001	3.61
Bilateral vlPFC	184	−38, 58, −6	0.001	3.19
	190	50, 40, −1	0.001	3.07

**p-values threshold: p < 0.005.**

### Exploratory Psycho-Physiological Interactions (PPI)

Psychophysiological interaction (PPI) (Friston et al., 1997; Friedman et al., 2017) a basic model of functional connectivity was used to explore network profiles of the functionally defined vmPFC seed during extinction learning. PPI models the response of target brain regions in terms of an interaction between a linear convolution of the physiological response of the *a priori* determined seed region and the psychological contrast of interest (e.g., CS+I > CS+U).

**FIGURE 4:**
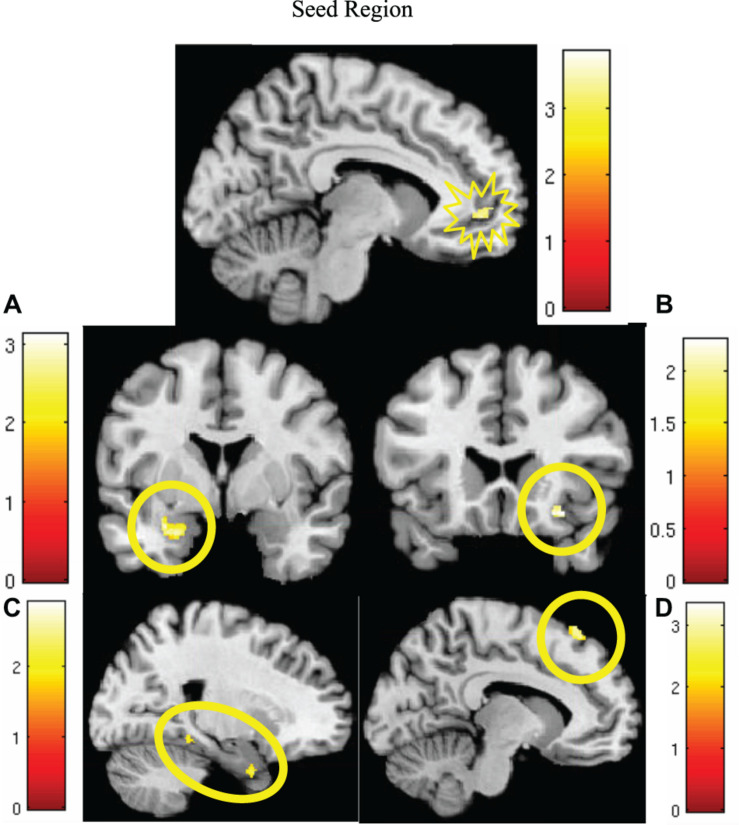
Significantly greater activity in the left vmPFC (top; red) for the instructed CS+ (CS+I) during extinction learning, and regions of significant co-activation. Regions of significant co-activation were identified using PPI, with the vmPFC as the seed (indicated within the starburst icon; top center). During extinction learning, significant co-activation of the **(A)** amygdala (middle left), **(B)** insula (middle right), **(C)** parahippocampus (bottom left), and **(D)** dmPFC (bottom right) was observed. Contrast: CS+I > CS+U for the early phase (first six trials) of extinction learning, MC corrected *p* < 0.05. Heat maps for each region represent Z-scores, which are reported alongside *p*-values in Table 4.

MCMC minimum cluster thresholds can be found in Table 2 for all bilateral masks, including those for which non-significant findings were not identified. The left vmPFC cluster derived from the activation results (CS+I > CS+U) and centered at the significance peak (coordinates: *x* = −10, *y* = 50, *z* = −1) was defined as the seed region For each participant, time series were extracted using the first eigenvariate of the weighted means of the modeled effects within a sphere (radius = 4 mm, *F* contrast, effects of interest, *p* < 0.99). This time series was convolved with the contrast of interest (CS+I > CS+U). The resultant PPI interaction terms models the effects of the vmPFC on any potential targets in the context of this psychological context (i.e., the contrast). Thus, each participant, contributed one first-level PPI map to a second level random effects analyses, to identify the modulatory effects of the vmPFC seed on the *a priori* network of regions (amygdala, insula, dACC, hippocampus, dmPFC, dlPFC, vlPFC, and vmPFC). Significant clusters (identified using a one-sample *t*-test) were observed in the dmPFC, parahippocampus, amygdala, and insula (Figure 4, statistical information in Table 4).

**TABLE 4:** Significant regions of co-activation with left vmPFC seed during PPI analysis, derived from ROI-based analyses.

*Early extinction, CS+I > CS+U*	Cluster size corrected in voxels	Coordinates	*p*-values (peak)	*Z*-scores
Bilateral parahippocampus	93	−10, −4, −19	0.006	2.51
	64	20, −38, −13	0.009	2.73
	36	21, 4, −31	0.012	2.57
Right insula	46	30, 22, −14	0.029	2.07
Left dmPFC	155	−12, 30, 59	0.003	2.79
Left amygdala	165	−22, −1, −24	0.004	2.65

**p-values threshold: p < 0.05.**

## Discussion

We open the Discussion by reiterating that this is the first study of the neurocircuitry evoked by the combined effects of instruction and experience on extinction learning. We reprise our results here. In assessing activation profiles for concurrently acquired fMRI data, we found increased activation in the vmPFC during the early phase of extinction learning when comparing the CS+I with CS+U, as well as increased dmPFC, vlPFC, and parahippocampal activity. PPI analysis motivated by our hypothesis and the activation results showed that the vmPFC seed significantly modulated the dmPFC, amygdala, insula, and parahippocampus during extinction learning. In the remainder of the paper, we discuss the importance of the results from the perspective of how extinction learning is represented in brain regions and networks, and conclude with implications of these results for clinical intervention.

### fMRI Effects: Activation and Network Profiles

The notably observed activation of the vmPFC is consistent with its purported role during extinction learning, social learning of safety, and cognitive reappraisal related emotion regulation. Previous studies have frequently reported vmPFC activation during extinction learning and its recall, and in general in response to safety signals (Milad et al., 2007b; Fullana et al., 2018). The vmPFC is also implicated in fear reversal studies when a CS previously associated with threat, is now safe. vmPFC is also suggested to have an inhibitory role on conditioned fear response during early extinction (for a review see Greco and Liberzon, 2016). Other works have shown involvement of the vmPFC in vicarious safety learning via social observation (Golkar et al., 2016) and reappraisal-related emotion regulation (Pico-Perez et al., 2019). A meta-analysis of a diverse group of emotion regulation tasks found vmPFC activation to be the most consistent element of negative emotion regulation whether via extinction learning, cognitive emotion regulation (reappraisal studies), or placebo effect (Diekhof et al., 2011). Previous work has shown that instruction about the absence of CS-US contingency leads to either immediate extinction (Mower, 1938; Rowles et al., 2012; Sevenster et al., 2012), or faster decay of the fear response (Koenig and Henriksen, 2005). Thus, our findings suggest that this facilitation may happen through increased activation in the vmPFC in conjunction with other areas involved in emotion regulation (dmPFC, parahippocampus, insula, and amygdala). Moreover, the PPI analyses suggest that the vmPFC exerts network modulation that reflects the network-based signatures of instruction. In this way, instruction-mediated emotion regulation may be the meta-process that underpins extinction learning. The application of PPI allows for our resultant findings to support this theory, given that PPI implies directionality.

Other related mPFC regions, vlPFC and dmPFC showed activation during instructed extinction learning. The dmPFC also showed significant coactivation with the vmPFC. Both the vlPFC and dmPFC are involved in emotional regulation, subsequent behavioral responding, and are also activated during threat appraisal (Milad et al., 2007a) and reappraisal (Buhle et al., 2014; Helion et al., 2019), which is a relevant mechanism involved here as a function of the instruction.

The parahippocampal cortex is both anatomically and functionally connected with the medial prefrontal cortex (Baldassano et al., 2013), and in addition to the hippocampus and vmPFC, is a key brain region involved in processing contextual associations (Aminoff et al., 2013). As previously noted, context plays an important role in signaling safety of the previously conditioned cues in the environment linked with extinction learning (Maren and Quirk, 2004). Activation of the parahippocampus, and its coactivation with the vmPFC, suggests a function of relaying safety instructions as a component of the cognitive context to indicate absence of the CS-US contingency (Javanbakht et al., 2017). Previous work has suggested that similar to the physical component of the context, instruction serves as a “cognitive context” that guides reactions when the conditions of the instruction are available (e.g., red light indicating absence of the loud noise) (Maren et al., 2013). Hippocampus and prefrontal cortex that are involved in context processing, are suggested to have a role in processing cognitive information during extinction learning (Garfinkel et al., 2014). In other words, cognitive context may be manipulated by the presentation of social cues or verbal instruction (Phelps et al., 2001). By changing the expectation, this cognitive manipulation can affect fear responses during extinction learning (Hugdahl and Ohman, 1977; Olsson and Phelps, 2004). This differs from cognitive “reappraisal,” as the participant is not instructed to *change/reappraise* salience of a cue, but rather is *informed* about the salience.

### Clinical Implications

These findings, albeit in healthy participants who were tested in an experimental lab setting, have implications for clinical practice. The finding of increased vmPFC activity during instructed extinction learning suggests that instruction may have an additive benefit in engaging this crucial structure associated with emotion and emotion regulation. Thus, therapeutic methods that utilize instruction may enhance efficacy in patients with anxiety disorder and PTSD by modulating the activity and network profiles of the vmPFC. For instance, evidence supports efficacy of both exposure therapy (based on experiential extinction learning), and cognitive processing therapy (focused on cognitive manipulation of patient’s perspective on the trauma leading to reduced fear) in treatment of PTSD (Cusack et al., 2016). However, there is a difference between laboratory models of fear conditioning and extinction learning and real clinical practice: Here fear learning happens in the laboratory and participants may give more credence to instruction provided by the experimenter, while in clinical practice fear learning has happened prior to engaging the therapist. While most laboratory models of exposure therapy are based on experiential extinction learning, such therapy involves a combination of experience and instruction. In the clinic a therapist is always signaling safety of the feared object via instruction. This is less applicable during generalization of extinction learning with self-practice outside of the clinic where the therapist is not available, although the memory of instruction is often still present. To realistically model the neurobiology of psychotherapy, we need to understand the combined role of the human social safety cue along with the experiential learning of safety. In this sense, our paradigm adds to the current laboratory models, to be more representative of the complexities of clinical work. Finally, understanding how instruction enhances extinction learning can help in individualized treatment and identifying those who may benefit from potential utilization of neuromodulation methods targeting these deficits in vmPFC to enhance response to therapy. Neuromodulation may be helpful as adjunct to therapy of conditions which have repeatedly shown deficits in vmPFC function, and which treatment involves cognitive behavioral therapy.

We also note some study limitations. The sample size for the fMRI analyses was relatively small (for reasons detailed in the section “Materials and Methods”), and the lack of viable psychophysiological data which is a general challenge (Bjorkstrand, 1990) also affected us. We were compelled to rely on self-reported expectancy as a measure of contingency awareness and successful conditioning, while noting that these measures have been shown to be associated with psychophysiological data (Indovina et al., 2011; Pohlack et al., 2012a,b). The “awareness” of fear is a clinically relevant patient experience, suggesting that contingency awareness may be an experimental surrogate of a clinically relevant “phenotype.” Additionally, multiple studies note that conditioned psychophysiological responses can be acquired even in the absence of cognitive awareness of contingency awareness (Ohman and Soares, 1994; Schultz and Helmstetter, 2010; Raio et al., 2012). Therefore, while our lack of viable psychophysiology data is certainly a limitation of this, our self-report expectancy and neuroimaging data still provide a valuable and meaningful contribution to the literature. As contingency awareness data was obtained at the end of the extinction learning phase, and not after the first six trials—early phase of extinction—we lack behavioral evidence of differences between CS+I and CS+U. Future studies should place an emphasis on gathering quality psychophysiological recordings and probe contingency awareness immediately after the early phase of extinction learning (first six trials), as well as at the end. Our neuroimaging data indicate greater recruitment of prefrontal and hippocampal areas when presented with CS+I compared to CS+U in the early phase of extinction, and these regions are typically associated with inhibition of conditioned fear responses. Therefore, we would expect to see differences in self-reported expectancy data after the first six trials, such that participants would report less expectancy of the US to follow the CS+I compared to the CS+U. However, this remains to be tested. Thus these data do not capture potential differences between CS+I and CS+U, suggesting that future studies should emphasize collection of physiological recordings *and* probe contingency awareness immediately after the early phase of extinction learning, and at the conclusion. Finally, while the formal bases of PPI permits the inference of directional (and putatively asymmetric) interactions between seeds and targets (Stephan, 2004), recovering the true bases of directionality in the brain is a fundamentally challenging question (Friston et al., 2012). The challenge is (a) empirical, given that the fMRI signal is limited by the hemodynamic filter that is stages removed from neuronal processes and (b) philosophical, given that discovering directionality is in part based on discovering “causality” (Mannino and Bressler, 2015). We submit that our claims of directionality refer to previous understanding of the involved networks, and in the narrow sense to the nature of inference based on PPI, and that we cannot make strong claims about general directionality in the brain.

## Data Availability Statement

The datasets presented in this article are not readily available although we may share the data on a case by case basis. Requests to access the datasets should be directed to AJ, ajavanba@med.wayne.edu.

## Ethics Statement

The studies involving human participants were reviewed and approved by the Wayne State University IRB. The patients/participants provided their written informed consent to participate in this study.

## Author Contributions

AJ has contributed to the design of the study, data analysis and interpretation, and writing the results. LG, SM, and AC contributed to the data collection, analysis, and interpretation, and wrote the results. IL contributed to the design of the study, data analysis, and interpretation, and wrote the results. VD contributed to the data analysis and interpretation, and wrote the results. All authors contributed to the article and approved the submitted version.

## Conflict of Interest

The authors declare that the research was conducted in the absence of any commercial or financial relationships that could be construed as a potential conflict of interest.
